# Cancercontribution: an innovative example of Participatory Medicine in cancer

**DOI:** 10.3332/ecancer.2011.ed9

**Published:** 2011-07-04

**Authors:** Giovanna Marsico, Silvia Camporesi

**Affiliations:** 1 Head of Project, Cancer Campus’ Pole Citoyen, Villejuif, Paris, France; 2 Centre for the Humanities & Health, King’s College London, UK

If we picked out the words most frequently used at the Health-care and social media Doctors 2.0 congress, ‘Participatory Medicine’ would stand out for sure together with ‘community’ and ‘empowerment’, building up a cluster of words around the concept of the evolving and more active role of the patient in medicine through social media and e-health tools. But what exactly is Participatory Medicine? And what are concrete and current examples of its implementation in Europe?

As defined by Gilles Frydman (Gilles, also at Doctors 2.0, is a pioneer of medical online communities and founder in 1995, of the Association of Cancer Online Resources, http://www.acor.org/), Participatory Medicine refers to a movement in which networked patients shift from being ‘mere passengers to responsible drivers of their health’. (http://participatorymedicine.org/)

While online social media are very powerful tools for enabling this shift, they are by no means the only ones. Indeed the concept itself of Participatory Medicine precedes, and is not limited to, social media online, as it encompasses a cooperative model of health care that encourages and expects active involvement by all connected parties (patients, caregivers, healthcare professionals, researchers, policy makers) as integral to the full continuum of care.

One innovative example of Participatory Medicine which relies on, but is not limited to, social media is the recently launched ‘Cancercontribution’, a project of collaborative intelligence based at the Villejuif Cancer Campus Pole Citoyen in Paris (http://www.cancercontribution.fr/). As the needs of society are constantly evolving due to the aging population, demographic changes, and a capillary diffusion of Information and Communication Technologies, the aim of Cancercontribution is to be proactive in this regard and provide a platform that, by involving first and foremost patients, caregivers and citizens, develops new participatory guidelines and political proposals to anticipate these changes.

Cancercontribution stimulates debates on sensible topics, such as opening up the news about a cancer diagnosis, working with cancer, the patients’ role in clinical trials, side effects of treatments, and so on. The platform invites the users-patients to express their opinions, following the formal principle that each opinion is given the same weight as the others.

The Cancercontribution agenda is based on three very fundamental points:

1. in chronic diseases, and especially in cancer (where the tendency to chronicisation is only going to increase in the coming decades due to demographic changes and to advances in therapies), patients demand more information than the evidence-based data offered by institutional actors;
2. An important and unique kind of expertise is brought forward by patients and caregivers’ experiences. This resource is unique in that it cannot be produced by classical experimental proceedings;
3. Expertise can be co-built by an interactive process which involves contributors of different backgrounds, such as patients, professionals, caregivers, politicians, associations, and civil society.

The collaborative expertise that is co-created by this interactive process constitutes ground to identify societal needs and health system’s weaknesses, while also leaving room for constantly improving existing practices. All the opinions and data collected are then analysed and elaborated to develop new participatory guidelines and political proposals. Such a ‘web laboratory’ is thus a tool that facilitates the exercise of citizenship and direct democracy, in so far as it gives space to each person’s opinion without the need of mediation through representatives, third parties or other associations.

It is in this regard that Cancercontributuon is a unique example of a laboratory that, while being grounded in the virtual world, goes beyond that to reach out and impact on the real one. As one of the key take-home messages from Doctors 2.0 was the need for an online space where doctors, patients and caregivers can communicate, exchange information, create and co-produce knowledge, the platform created by Cancercontribution can be regarded as one important step in this direction for cancer care in Europe within the framework of Participatory Medicine.

